# Ablation of TSC2 Enhances Insulin Secretion by Increasing the Number of Mitochondria through Activation of mTORC1

**DOI:** 10.1371/journal.pone.0023238

**Published:** 2011-08-19

**Authors:** Maki Koyanagi, Shun-ichiro Asahara, Tomokazu Matsuda, Naoko Hashimoto, Yutaka Shigeyama, Yuki Shibutani, Ayumi Kanno, Megumi Fuchita, Tomoko Mikami, Tetsutya Hosooka, Hiroshi Inoue, Michihiro Matsumoto, Masato Koike, Yasuo Uchiyama, Tetsuo Noda, Susumu Seino, Masato Kasuga, Yoshiaki Kido

**Affiliations:** 1 Division of Diabetes and Endocrinology, Kobe University Graduate School of Medicine, Kobe, Japan; 2 Division of Cellular and Molecular Medicine, Kobe University Graduate School of Medicine, Kobe, Japan; 3 Kobe University Graduate School of Health Sciences, Kobe, Japan; 4 Frontier Science Organization, Kanazawa University, Kanazawa, Japan; 5 Department of Cell Biology and Neurosciences, Juntendo University Graduate School of Medicine, Tokyo, Japan; 6 Department of Cell Biology, Cancer Institute, Japanese Foundation of Cancer Research, Tokyo, Japan; 7 Research Institute National Center for Global Health and Medicine, Tokyo, Japan; University of Windsor, Canada

## Abstract

**Aim:**

We previously found that chronic tuberous sclerosis protein 2 (TSC2) deletion induces activation of mammalian target of rapamycin Complex 1 (mTORC1) and leads to hypertrophy of pancreatic beta cells from pancreatic beta cell-specific TSC2 knockout (βTSC2^−/−^) mice. The present study examines the effects of TSC2 ablation on insulin secretion from pancreatic beta cells.

**Methods:**

Isolated islets from βTSC2^−/−^ mice and TSC2 knockdown insulin 1 (INS-1) insulinoma cells treated with small interfering ribonucleic acid were used to investigate insulin secretion, ATP content and the expression of mitochondrial genes.

**Results:**

Activation of mTORC1 increased mitochondrial DNA expression, mitochondrial density and ATP production in pancreatic beta cells of βTSC2^−/−^ mice. In TSC2 knockdown INS-1 cells, mitochondrial DNA expression, mitochondrial density and ATP production were increased compared with those in control INS-1 cells, consistent with the phenotype of βTSC2^−/−^ mice. TSC2 knockdown INS-1 cells also exhibited augmented insulin secretory response to glucose. Rapamycin inhibited mitochondrial DNA expression and ATP production as well as insulin secretion in response to glucose. Thus, βTSC2^−/−^ mice exhibit hyperinsulinemia due to an increase in the number of mitochondria as well as enlargement of individual beta cells via activation of mTORC1.

**Conclusion:**

Activation of mTORC1 by TSC2 ablation increases mitochondrial biogenesis and enhances insulin secretion from pancreatic beta cells.

## Introduction

Type 2 diabetes mellitus is characterised by insulin resistance in peripheral tissues and pancreatic beta cell failure. Impairment of insulin secretory capacity has been shown to contribute to the onset of type 2 diabetes. It remains controversial whether such impairment of insulin secretory capacity is caused by an impairment of the functions of pancreatic beta cells alone, whether it occurs as a result of a decrease in pancreatic beta cell mass alone, or whether both factors are involved and influence each other [1]–[4]. It is therefore important to clarify the mechanism of the impairment of insulin secretory capacity in order to elucidate the mechanism of pathogenesis of type 2 diabetes.

Pancreatic beta cells sense ambient glucose concentrations, and various kinds of metabolites resulting from glucose metabolism in pancreatic beta cells, such as ATP, are important not only as energy sources for cells but also as signals for inducing insulin secretion [5]. Insulin secretion from pancreatic beta cells not only maintains appropriate blood glucose levels, but also plays an important role in maintaining the functions of the beta cells themselves [6], [7]. Glucose is metabolised into pyruvic acid via glycolysis in the pancreatic beta cells, and pyruvic acid enters the tricarboxylic acid cycle and is oxidised to CO_2_ and H_2_O with the generation of ATP in mitochondria [8]. It has been reported that no glucose-responsive insulin secretion is found in MIN6 cells depleted of mitochondrial DNA [9]. In addition, decreased insulin secretory capacity, decreased ATP production and abnormalities in mitochondrial morphology have been found in isolated islets from mouse models of diabetes and patients with type 2 diabetes [10], [11]. Thus, mitochondria are clearly associated with the cellular functions of pancreatic beta cells.

We have previously shown that the insulin signalling pathway is responsible for regulation of both the number and size of pancreatic beta cells in mice [6], [12]. Tuberous sclerosis is an autosomal dominant disorder characterised by formation of hamartomas. The genes for tuberous sclerosis complex (TSC) 1 and TSC2 have been identified as causative genes of inherited TSC [13], [14]. Akt-mediated multiple phosphorylation of TSC2 inhibits its ability to act as a GTPase-activating protein toward Rheb, resulting in activation of mTOR complex 1 (mTORC1) [15]. Previously, we found that pancreatic beta cell–specific TSC2 knockout (βTSC2^−/−^) mice, in which mTORC1 is constitutively active, exhibited an increase in the size of individual beta cells and a decrease in the number of beta cells via a negative feedback mechanism [12]. Alongside this role in the regulation of pancreatic beta cell mass, we also found that ablation of TSC2 in pancreatic beta cells may augment the ability to secrete insulin [12].

The above results prompted us to consider that mTORC1 might regulate not only pancreatic beta cell mass but also insulin secretion, and therefore in the present study we analysed the islets of pancreatic beta cell-specific TSC2 knockout (βTSC2^−/−^) mice and a TSC2 knockdown beta cell line. Here we succeeded in showing that constitutive activation of mTORC1 enhances insulin secretion by increasing the number of mitochondria.

## Methods

### Mice

We generated heterozygous pancreatic beta cell–specific TSC2 knockout (βTSC2^+/−^) mice by crossing *TSC2^flox/flox^* mice [12] with those that express the *Cre* recombinase gene under the control of the rat insulin-2 gene [16] as described previously [12]. Animals were maintained on a 12 h light, 12 h dark cycle and fed normal chow from the time of weaning (3 weeks old), as described [17], [18]. This study was performed in accordance with the guidelines of the Animal Ethics Committee of Kobe University Graduate School of Medicine.

### Cell culture and transfection of siRNA

At 24 h before transfection, INS-1 cells were re-plated in 12 well plates (60 mm dishes) and transfected with small interfering RNA (siRNA) for TSC2 (SMARTpool; Dharmacon, Lafayette, CO, USA) or scramble controls (Non-Targeting siRNA#2; Dharmacon) with DharmaFECT2 transfection reagent (Dharmacon). After a further incubation of 24 h for mRNA or 72 h for protein, the cells were harvested for evaluation of insulin secretion and mRNA or protein expression.

### Assay of insulin secretion from isolated islets

Islets were isolated from 8-week-old mice as described previously [18], [19]. To assay insulin release, 5 islets were manually selected, incubated in Krebs-Ringer solution and stimulated at 37°C with various concentrations of either glucose for 1 h or KCl for 30 min. The islets were then collected by centrifugation, and the supernatant was assayed for insulin secretion by an ELISA kit with a mouse insulin standard (Shibayagi Co., Gunma, Japan). For measurement of islet insulin content, islets were solubilised in acid-ethanol solution (74% ethanol, 1.4% HCl) overnight at 4°C before insulin ELISA.

Insulin secretion from INS-1 cells was measured after a 30 min incubation in Krebs-Ringer-bicarbonate-4-(2-hydroxyethyl)-1-piperazine ethanesulfonic acid (HEPES) buffer (KRBH; 140 mM NaCl, 3.6 mM KCl, 0.5 mM NaH_2_PO_4_, 0.5 mM MgSO_4_, 1.5 mM CaCl_2_, 2 mM NaHCO_3_, 10 mM HEPES, and 0.1% BSA) containing the indicated stimulators. The insulin content was determined after extraction with acid ethanol.

### ATP content

Isolated islets derived from 8-week-old mice were incubated with 2.8 mM and 11.2 mM glucose for 1 h after a 30 min starvation. ATP levels were measured with Cellno ATP Assay Kit Type N (TOYO BNet Co., Ltd, Tokyo, Japan). Luminescence of an aliquot of the solution was measured with a luminometer.

INS-1 cells were plated in a 96 well microtest tissue culture plate (35-3072; BD Falcon, Franklin Lakes, NJ, USA). The next day, siRNAs for TSC2 and scramble controls were transfected into the cells. After 48 h, ATP levels were determined using a ‘Cellno’ ATP Assay Kit Type N (TOYO BNet) according to the manufacturer's instructions. Briefly, 100 µL of the lysis/assay solution provided by the manufacturer was added to the cells. After shaking for 1 min and incubating for 10 min at 23°C, luminescence of an aliquot of the solution was measured in a luminometer.

### Real-time RT-PCR analysis

Total cellular RNA was extracted from islets isolated from control and βTSC2^−/−^ mice and INS-1 cells with an RNeasy kit (QIAGEN, Valencia, CA, USA). Real-time RT-PCR analysis of the total RNA pooled from 6 animals of each genotype was performed as described previously [20]. For real-time quantitative reverse transcription and PCR analysis, cDNA synthesized from total RNA was evaluated in a sequence detector (model 7900; Applied Biosystems, Foster City, CA, USA) with specific primers and SYBR Green PCR Master Mix (Applied Biosystems). The relative abundance of mRNAs was calculated with *cyclophilin* mRNA as the invariant control. Details of the primers used for RT-PCR can be found in Table S1.

### MitoTracker Red staining

After washing the cells with phosphate buffered saline, INS-1 cells were stained with 20 nM MitoTracker Red (Invitrogen, Carlsbad, CA, USA) and subjected to fluorescence-activated cell sorting (FACS) analysis. A minimum of 10,000 cells were analyzed on a BD FACSCalibur cell sorter (BD Biosciences, San Jose, CA, USA) using Cell Quest software (BD Biosciences). The mean fluorescence intensity was plotted for each treatment.

### Electron microscopy

Two *TSC2^flox/flox^* and two βTSC2^−/−^ mice were anesthetised with pentobarbital (25 mg/kg, i.p.) and subjected to cardiac perfusion with 2% glutaraldehyde and 2% paraformaldehyde buffered with 0.1 M sodium phosphate buffer (pH 7.2). The pancreas was excised from each mouse, cut into small pieces and immersed overnight in the same fixative. The tissue was then postfixed with 2% osmium tetroxide, block-stained in 1% uranyl acetate, dehydrated with a graded series of ethanol and embedded in Epon812 (TAAB). Thin sections were stained with uranyl acetate and lead citrate before examination under an electron microscope (7100; Hitachi, Tokyo, Japan). For light microscopy, 1 µm–thick sections were cut and stained with toluidine blue.

### Morphometry

Morphometric analysis of the mitochondria in pancreatic beta cells was performed as described previously [21]. Briefly, electron micrographs of pancreatic beta cells were taken at a primary magnification of ×5000 (n = 23 for control and n = 21 for βTSC2^−/−^ mice). After printing at ×2.9 the original magnification on projection papers, we estimated the mitochondrial density volume by point counting, using a double-lattice test system with a 1.5 cm spacing. The volume density (Vv) of mitochondria was expressed as percent volume: Vv = (Pi/Pt)×100 (%), where Pi is the number of points falling on each mitochondrial structure and Pt is the number of points falling on the cytoplasm of pancreatic beta cells.

### Immunoblot analysis

We prepared lysates of isolated islets or INS-1 cells as described [17], [18]. The lysates were probed with antibodies to TSC2, Akt, the phospho-Thr^308^ form of Akt, the phospho-Ser^473^ form of Akt, p70 S6 kinase, the phospho-Thr^389^ form of p70 S6 kinase, S6, the phospho-Ser^235^ and -Ser^236^ forms of S6, voltage-dependent anion-selective channel (VDAC) protein and cytochrome c oxidase complex IV (COXIV; all from Cell Signaling, Danvers, MA, USA). Antibodies to SDH (from Abcam, Cambridge, UK) as well as that to β-actin (from Sigma-Aldrich, St. Louis, MO, USA) were also used.

### Statistical analysis

Data are presented as means ± standard errors of the means. We assessed the significance of differences between independent means by the Student's *t*-test. A *P* value of <0.05 was considered statistically significant.

## Results

### Insulin secretory response to glucose was enhanced in islets of βTSC2^−/−^ mice

**Figure 1:**
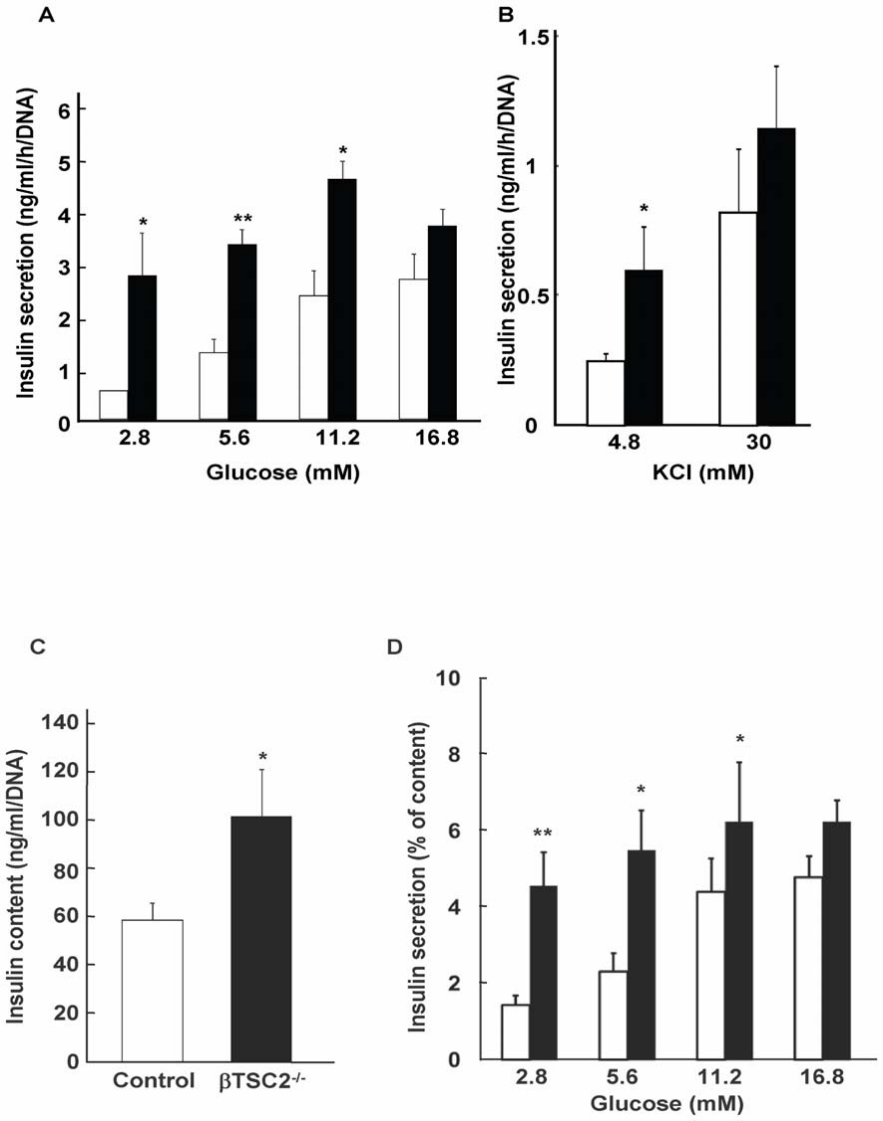
Effect of beta cell–specific ablation of TSC2 on insulin release and insulin content. (**A** and **B**) Insulin release in response to the indicated concentrations of glucose (**A**) or KCl (**B**) was measured with islets isolated from control (white bars) or βTSC2^−/−^ (black bars) mice at the age of 8 weeks. Data were obtained from four independent experiments (*n* = 4–6). (**C**) Insulin content of isolated islets at the age of 8 weeks. (**D**) The amount of insulin secreted from the isolated islets normalised to insulin content. Data were obtained from two to three independent experiments (*n* = 4–6). All data are shown as means±SE of values from 4 animals of each genotype. *P<0.05, **P<0.01 vs the corresponding value for control mice.

As previously described [12], βTSC2^−/−^ mice show hypoglycemia and hyperinsulinemia at young ages. The insulin responses to glucose are also abnormal in βTSC2^−/−^ mice, with the plasma insulin levels after glucose challenge markedly higher than those in control mice [12]. In the present study, we further examined glucose-stimulated insulin secretion in isolated islets from control and βTSC2^−/−^ mice at 8 weeks of age. Glucose induced a concentration-dependent increase in insulin secretion from control islets in static incubations (Fig. 1A). In contrast, the insulin response of islets from βTSC2^−/−^ mice to glucose was significantly greater than that of control islets at both low and high glucose concentrations, except at 16.8 mM glucose, which showed no significant difference (Fig. 1A). The islets of βTSC2^−/−^ mice exhibited a normal insulin secretory response to a high concentration of KCl (Fig. 1B), which elicits insulin release by inducing membrane depolarisation and calcium influx. Because βTSC2^−/−^ mice have shown an increase in the individual size of beta cells, the enhanced insulin secretion may be attributable to increased insulin synthesis. The total insulin content of βTSC2^−/−^ mice was significantly higher than that of control mice by ∼1.5-fold (Fig. 1C). We therefore evaluated the insulin response to glucose normalised to insulin content. After normalisation, the insulin secretory response to glucose in βTSC2^−/−^ mice was still significantly higher than that in control mice (Fig. 1D), suggesting that a factor in addition to enhanced insulin synthesis is involved in the increased insulin response to glucose in islets of βTSC2^−/−^ mice.

The insulin secretory response of islets from βTSC2^−/−^ mice was significantly greater than that of control mice at low glucose concentrations (Fig. 1A and D), suggesting high sensitivity to glucose in pancreatic beta cells.

### Number of mitochondria was increased in islets of βTSC2^−/−^ mice

**Figure 2:**
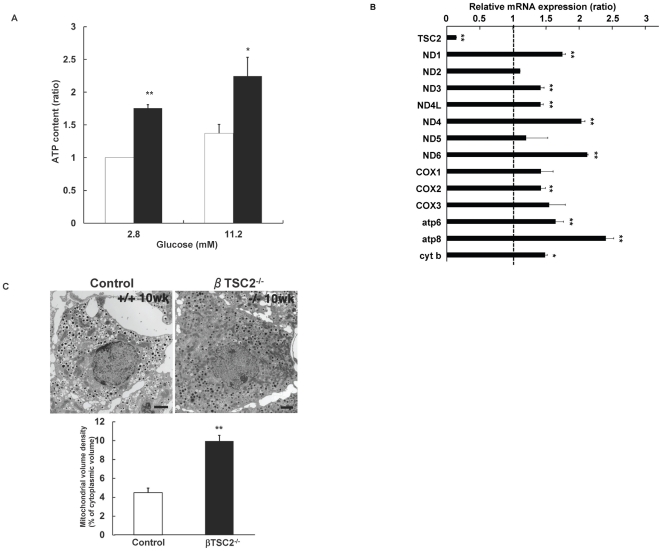
Effect of beta cell–specific ablation of TSC2 on mitochondrial function in pancreatic beta cells. (**A**) ATP content of isolated islets of control and βTSC2^−/−^ mice at 8 weeks of age. Data are shown as means±SE from 4 mice per each genotype. Data were obtained from three independent experiments. *P<0.05, **P<0.01 vs the corresponding value for control mice. (**B**) The abundance of mRNAs for the indicated proteins was determined by real-time RT-PCR analysis of total RNA isolated from islets of control and βTSC2^−/−^ mice. The amounts of the mRNAs in βTSC2^−/−^ mice are expressed relative to those in control animals. Data are means±SE of triplicates for pooled total RNA samples from 3 mice of each genotype. Data were obtained from four independent experiments. *P<0.05, **P<0.01 vs the corresponding value (1.0) for control mice. (C) Upper panel: Electron micrograph of beta cells of control and βTSC2^−/−^ mice at 10 weeks of age. Scale bar: 1 µm. Lower panel: The volume density (%) of mitochondria in pancreatic beta cells obtained from control and βTSC2^−/−^ mice at 10 weeks of age.

We next examined whether enhanced activity of mTORC1 resulted in an elevation of the ratio of ATP to ADP, which induced closure of the ATP-sensitive K-channels, depolarisation of the beta cell plasma membrane and influx of Ca^2+^ via voltage-dependent Ca-channels, leading to insulin secretion. ATP production was increased in the islets of βTSC2^−/−^ mice at 8 weeks of age under physiologic condition (11.2 mM glucose) and low glucose concentration (2.8 mM glucose) (Fig. 2A). We then measured mRNA expression of all 13 genes encoded in the mitochondria, comprising 7 subunits of complex I (NADH dehydrogenase; ND1 to ND6), 1 subunit of complex III (cytochrome c oxidoreductase; cyt b), 3 subunits of complex IV (cytochrome c oxidase; COX1 to COX3) and 2 subunits of complex V (ATP synthase; atp6 and atp8). Mitochondrial DNA expression was significantly greater in islets of βTSC2^−/−^ mice than in control mice (Fig. 2B). Electron microscopy revealed that while the morphology of mitochondria was not apparently altered in pancreatic β cells of either genotype, the volume density of mitochondria in pancreatic beta cells of βTSC2^−/−^ mice was significantly increased (about 2.6-fold) compared with that of control mice at 10 weeks of age (Fig. 2C). These results suggest that the increased number of mitochondria resulted in the augmented ATP production in the pancreatic beta cells of βTSC2^−/−^ mice.

### TSC2 knockdown INS-1 cells led to enhancement of insulin secretion

**Figure 3:**
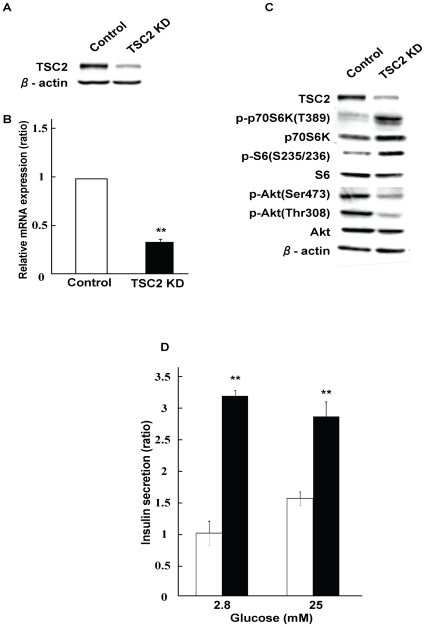
Establishment of TSC2 knockdown INS-1 cells. (**A**, **B** and **C**) INS-1 cells treated with scramble siRNA (control) and TSC2 siRNA (ΔTSC2) were lysed and subjected to immunoblot analysis with antibodies against TSC2 (**A**) or the indicated proteins (**C**), or subjected to real-time PCR analysis of *Tsc2* mRNA (**B**). Data in **B** are relative expression values for INS-1 cells treated with scramble siRNA (control) and are means±SE from four independent experiments. **P<0.01 (**D**) Insulin secretion in response to the indicated concentrations of glucose for 30 min was assessed in INS-1 cells and expressed per DNA content. Data were obtained from four independent experiments.

To further confirm the phenotype of βTSC2^−/−^ mice described above, we established TSC2 knockdown INS-1 cells. TSC2 was knocked down with siRNA. TSC2 protein and *Tsc2* mRNA expression were significantly reduced by approximately 75% at the protein level and 70% at the mRNA level (Fig. 3A and B). Reduction in TSC2 expression resulted in an increase in the phosphorylation of p70 S6 kinase and S6 ribosomal protein, together with a decrease in the phosphorylation of Akt at Thr^308^ and Ser^473^ (Fig. 3C). These results indicated that mTORC1 was activated by depletion of TSC2 and that activated mTORC1 downregulated Akt phosphorylation via a negative feedback system, which is consistent with the data obtained from analysis of βTSC2^−/−^ mice [12].

We performed a glucose-stimulated insulin release assay with TSC2 knockdown INS-1 cells. TSC2 knockdown INS-1 cells exhibited a significant increase in insulin secretion at both low (2.8 mM) and high (25 mM) glucose concentrations (Fig. 3D). The insulin secretory response to low glucose concentrations was at a maximum without further augmentation of insulin secretion at higher glucose concentrations. These results indicate that reduction of TSC2 expression induced the activation of mTORC1, leading to enhancement of insulin secretion in vitro as well as in vivo as shown above.

### TSC2 knockdown augmented mitochondrial number and function in pancreatic beta cell line cells

**Figure 4:**
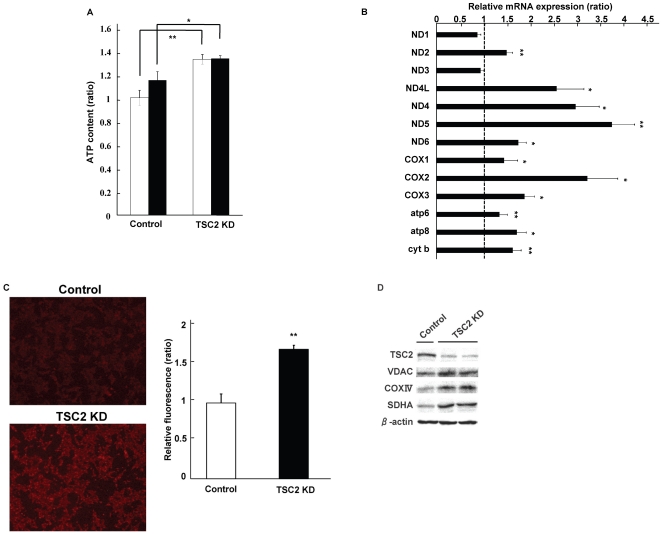
Effect of TSC2 knockdown on the function of mitochondria from INS-1 cells. (**A**) ATP content of control and TSC2. Cellular ATP level was measured at 2.8 mM (white bar) and 25 mM (black bar) glucose (*n* = 12). The figure shows data from one representative of four independent experiments. (**B**) The level of mRNA expression of mitochondria DNA-encoded genes obtained by real-time PCR. Data are means ± SE of triplicate for pooled total RNA samples from three independent experiments. The amounts of the mRNAs in TSC2 INS-1 cells are expressed relative to those in control cells. (**C**) Mitochondrial density measured as described in the Material and Methods with MitoTracker Red. Left panel: Mitochondria in control cells and TSC2 INS-1 cells were stained. Right panel: Relative fluorescence was measured by fluorescence-activated cell sorting analysis after MitoTracker Red staining. Data were obtained from three independent experiments. (**D**) Mitochondrial protein expression assessed by western blotting in INS-1 cells.

We next examined the effect of TSC2 knockdown on the function of mitochondria from INS-1 cells. Reduction of TSC2 expression significantly increased ATP production in INS-1 cells (Fig. 4A). There was a significant difference in ATP production between TSC2-knockdown and control INS-1 cells both at 2.8 and 25 mM. High glucose concentrations did not induce further large increases in glucose-stimulated insulin secretion and ATP production compared with those at low glucose concentrations, possibly because they are greatly augmented even at low glucose concentrations in TSC2-knockdown INS-1 cells. We next examined the effect of TSC2 knockdown on the expression of mitochondrial DNA by real-time RT-PCR. Expression of mRNA by mitochondrial DNA was increased in TSC2 knockdown INS-1 cells, consistent with the results obtained in the islets of βTSC2^−/−^ mice (Fig. 4B). We also measured the amount of mitochondria with MitoTracker Red, which is a fluorescent dye that stains the mitochondrial membrane. Staining of the mitochondrial membrane in TSC2 knockdown INS-1 cells was about 1.7-fold more intense than in control INS-1 cells, suggesting an increase in the number of mitochondria (Fig. 4C). We also examined the expression of proteins comprising the mitochondrial membrane by immunoblot analysis. VDAC, COXIV and succinate dehydrogenase complex subunit A are mitochondrial membrane proteins encoded in the nucleus. The expression of these proteins was increased in TSC2 knockdown INS-1 cells (Fig. 4D). These results indicate that reduced expression of TSC2 induced an increase in the number of mitochondria and ATP production in vitro as well as in vivo.

### Rapamycin treatment inhibited enhanced mitochondrial function in INS-1 cells

**Figure 5:**
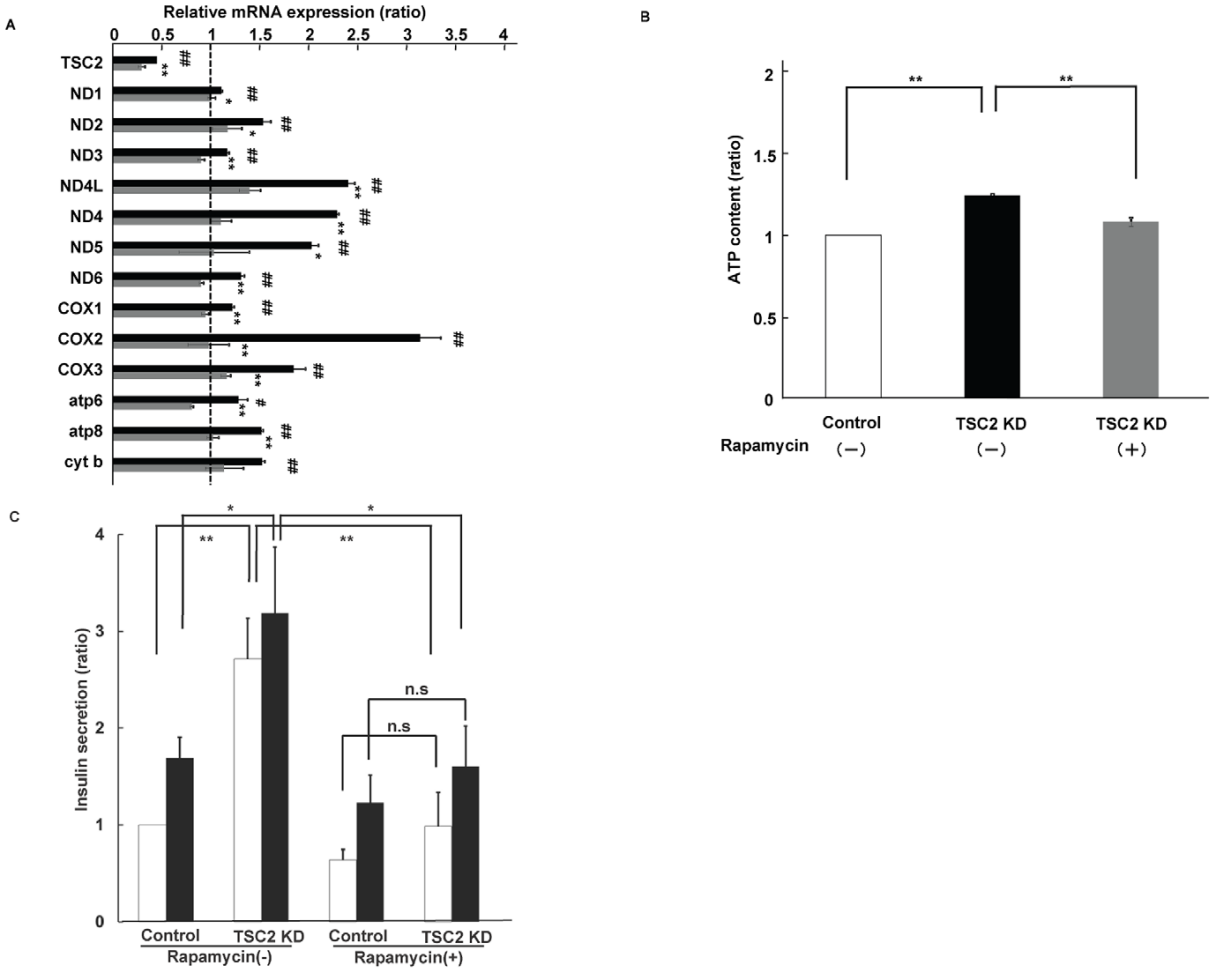
Effects of rapamycin treatment on mitochondrial function in TSC2 INS-1 cells. (**A**) Effect of rapamycin treatment on mitochondrial DNA expression. The ratio of TSC2 INS-1 cells untreated (black bar) and treated (grey bar) with 20 nM rapamycin to control cells. Data are means ± SE of triplicate pooled total RNA samples from three independent experiments. #*P*<0.05; ##*P*<0.01 TSC2 cells compared with control (vehicle) cells. *P<0.05; **P<0.01 TSC2 cells treated with rapamycin compared with TSC2 INS-1 cells treated with vehicle. (**B**) Analysis of rapamycin effect on ATP production. ATP content of control cells (open bar), TSC2 INS-1 cells treated with (grey bar) and without (lack bar) 20 nM rapamycin (*n* = 12) Figure shows data from one representative of two independent experiments. (**C**) Insulin secretion in response to 2.8 mM (open bar) and 25 mM (filled bar) glucose for 30 min was assessed in INS-1 cells and expressed per DNA content. Data are shown with and without 20 nM rapamycin (*n* = 6). Figure shows data from one representative of three independent experiments.

To further determine whether enhanced mTORC1 activity is responsible for the increase in mitochondrial biogenesis, we treated TSC2 knockdown INS-1 cells with rapamycin, an mTORC1 inhibitor. Treatment with 20 nM rapamycin reduced the expression levels of mitochondrial DNA to control levels in TSC2 knockdown INS-1 cells (Fig. 5A). The amount of ATP produced and the insulin secretory response to glucose were also reduced to the levels in control cells with rapamycin treatment (Fig. 5B and C). These results suggest that enhanced mTORC1 activity might induce an increase in mitochondrial density that increases ATP production and promotes insulin secretion.

## Discussion

Insulin-secreting pancreatic beta cells use their insulin receptors to transmit insulin signals into the cells. We previously found that the insulin signalling pathway in pancreatic beta cells is essential for maintenance of pancreatic beta cell mass [6]. It has been shown that constitutive activation of mTORC1 by inhibiting the function of TSC2 is involved in the control of protein synthesis, cell proliferation, intracellular nourishment and regulation of transcription factors [15], [22]–[25]. We have recently shown that pancreatic beta cell–specific TSC2 knockout (βTSC2^−/−^) mice exhibit increased pancreatic beta cell mass due to constitutive enhancement of mTORC1 activity [12]. In the present study, we found in pancreatic beta cells that mTORC1 hyperactivity, which results from inhibition of TSC2 expression, enhanced insulin secretion by increasing the number of mitochondria.

The 13 types of proteins encoded by mitochondrial DNA are important subunits of the respiratory chain complexes (I, III, IV and V), which produce most of the energy required for cellular activity [26]. Mitochondria are also important to ATP production in pancreatic beta cells, and the pathogenesis of mitochondrial diabetes involves pancreatic beta cell failure resulting from mitochondrial DNA mutations [27]. This study showed that the pancreatic islets of βTSC2^−/−^ mice exhibited increased mitochondrial DNA content and an increased number of mitochondria, as determined by electron microscopy. This increase in the number of mitochondria is expected to enhance the insulin secretory capacity by increased ATP production. In fact, we found that the phenotype of TSC2 knockdown INS-1 cells was restored by administration of the mTORC1 inhibitor, rapamycin. Thus, these findings indicate that mTORC1 hyperactivity in pancreatic beta cells increased the insulin secretory response to glucose via an increase in the number of mitochondria.

Peroxisome proliferator-activated receptor gamma coactivator 1-alpha (PGC1α) was the first factor discovered in regulation of the biosynthesis of mitochondria [28], [29]. PGC1α is a coactivator of the transcription factor nuclear respiratory factor 1 (NRF)-1/2, and NRF-1/2 induces transcription and stabilisation of mitochondrial DNA by activating transcription factor A, mitochondrial (TFAM), which is encoded in the nucleus [28], [30]–[32]. Mice lacking TFAM specifically in pancreatic beta cells have decreased insulin secretory capacity associated with a depletion of mitochondrial DNA and abnormalities in mitochondrial morphology [33]. They become diabetic at 5 weeks of age, and their beta cell mass is found to decrease with age. AMP-activated protein kinase (AMPK) is known to be involved in insulin secretion and cell survival in pancreatic beta cells [34] and is also another important factor that regulates the biosynthesis of mitochondria [35], [36]. Aminoimidazole carboxamide ribonucleotide, which activates AMPK, stimulates the biosynthesis of mitochondria through PGC1α and NRF [37], [38].

Previous studies in HEK293 cells have shown that mitochondrial functions are enhanced by mTOR, which regulates cell growth and proliferation according to the nutritional environment [39]. Recently, it has also been found that rapamycin inhibits the transcription of mitochondrial genes by dissociating PGC1α from the complex of TORC1 and the transcription factor YY1 [40]. The facts that PGC1α functions as a coactivator of YY1, that the YY1-PGC1α complex is necessary for the expression of mitochondrial genes and that the binding of the complex is dependent on TORC1 activity indicate that mitochondrial function is influenced by the nutritional environment and by growth factors through mTORC1 activity. This study also showed that expression levels of mRNA for mitochondrial DNA were enhanced in INS-1 cells in which TSC2 expression was inhibited by siRNA and were inhibited by administration of rapamycin, suggesting that rapamycin inhibited the transcription of mitochondrial DNA by dissociating the binding between PGC1α and YY1.

As mentioned above, the expression of mitochondrial genes is known to be controlled by PGC1α, but no change in the expression level of PGC1α was observed in TSC2-knockdown INS-1 cells (data not shown). Morino et al. reported a decrease in mitochondrial density and mitochondrial proteins in the skeletal muscles of patients with type 2 diabetes mellitus, but found no difference between the expression levels of PGC1α, NRF and TFAM [41]. Recently, it has been reported that the activity of PGC1α requires not only its expression but also modification of processes such as deacetylation and phosphorylation [42]. After being deacetylated by Sirt1 or phosphorylated AMPK, PGC1α functions as a co-activator of transcription factors [43], [44]. As deletion of TSC2 increases AMPK phosphorylation [45], [46], we confirmed that the phosphorylation of AMPK was enhanced in the pancreatic islets of βTSC2^−/−^ mice (data not shown). In the pancreatic islets of βTSC2^−/−^ mice, phosphorylated AMPK might also increase the number of mitochondria through activation of PGC1α.

Young βTSC2^−/−^ mice have greater pancreatic beta cell mass and increased secretion of insulin by individual beta cells. We previously reported that in older βTSC2^−/−^ mice, constitutive hyperactivity of mTORC1 induces a negative feedback mechanism and attenuation of insulin signalling that results in decreased pancreatic beta cell mass and development of diabetes mellitus [12]. In addition to attenuation of insulin signalling, we recently found that excessive insulin secretion induces endoplasmic reticulum (ER) stress [47]. It is possible, therefore, that hyperinsulinemia, which is also found in young βTSC2^−/−^ mice, may promote ER stress, leading to apoptosis of pancreatic beta cells and resulting in reduced pancreatic beta cell mass in older mice. In addition, it has been reported that constitutively enhanced mTORC1 activity induces ER stress [48]. That study, in which mouse embryonic fibroblasts were used, indicated that the deletion of TSC2 increases the likelihood of ER stress-induced apoptosis when mTORC1 activity is enhanced. Thus, in βTSC2^−/−^ mice, attenuation of insulin signalling mediated by a negative feedback mechanism and ER stress due to excessive insulin secretion are thought to overlap and contribute to a decrease in pancreatic beta cell mass in older mice.

We have shown that constitutive activation of mTORC1 enhances insulin secretion by increasing the number of mitochondria. As seen in the phenotype of βTSC2^−/−^ mice, constitutive hyperactivity of mTORC1 resulted in decreased pancreatic beta cell mass. Thus, it is suggested that moderate mTORC1 activity is required to preserve the pancreatic beta cell mass and maintain the insulin secretory capacity of pancreatic beta cells. As mentioned above, mTORC1 activity is thought to be involved in the pathogenesis of type 2 diabetes mellitus through regulation of the cellular functions of pancreatic beta cells by control of the number of mitochondria. Further studies of the mechanism of regulation of mitochondrial functions in pancreatic beta cells are required for the prevention and treatment of type 2 diabetes mellitus.

## Supporting Information

Table S1 **Primers used for real-time RT-PCR analysis.**
(DOC)
