# Supplementary material for: Ablation of TSC2 Enhances Insulin Secretion by Increasing the Number of Mitochondria through Activation of mTORC1
Source: PLoS One. 2011 Aug 19;6(8):e23238. doi: 10.1371/journal.pone.0023238 (PMC3158755; doi:10.1371/journal.pone.0023238)
Supplement: Table S1 — Primers used for real-time RT-PCR analysis. (DOC) [file pone.0023238.s001.doc]

Supplementary Table 1. Primers used for real-time RT-PCR analysis

| **Gene** | **Sense** | **Antisense** |
| --- | --- | --- |
| Mouse | | |
| *TSC2* | 5-CCGCAACCTGTCCTTTGTGG -3′ | 5′- AATGTGGCGGAGTCTTGCGA -3′ |
| *ND1* | 5-CGCCCTAACAACTATTATCTTCC -3′ | 5′-GAAGCGTGGATAAGATGCTC -3′ |
| *ND2* | 5′-GGCCTTCCACCACTAACAGG -3′ | 5′-AGGGTGGAAAATATTAGGTTGGGT -3′ |
| *ND3* | 5′-ACAAGCTCTGCACGTCTACC -3′ | 5′- GCTCATGGTAGTGGAAGTAGAAGAG-3′ |
| *ND4L* | 5′-CCATACCAATCCCCATCACC -3′ | 5′- CGTAATCTGTTCCGTACGTGTT-3′ |
| *ND4* | 5′- TTAACCTCCAACCCTCACAC-3′ | 5′- AGGCCTGTAATTAGTTTTGGAC-3′ |
| *ND5* | 5′- ACCCATAAAATCTCTCAACC-3′ | 5′-GTGGTTATGTTTGTGTGAAG -3′ |
| *ND6* | 5′- AAAACGATCCACCAAACCCT-3′ | 5′- GGTTAGCATTAAAGCCTTCACC-3′ |
| *COX1* | 5′-CCTTTGCTTCAAAACGAGAA -3′ | 5′-ATAGGTTGGTTCCTCGAATG -3′ |
| *COX2* | 5′-CAAGCAACAGTAACATCAAACC-3′ | 5′-GTGGAACCATTTCTAGGACAA -3′ |
| *COX3* | 5′- TGTTTGCCTACTACGACAAC-3′ | 5′-GGAAAAGTCAGACTACGTCTAC -3′ |
| *atp6* | 5′-AAATATTAGCCCACCAACAG -3′ | 5′-CTAGGAGGGTGAATACGTAG -3′ |
| *atp8* | 5′-AGTCTCATCACAAACATTCCC -3′ | 5′-GTTAGTGATTTTGGTGAAGGTG -3′ |
| *cytob* | 5′-GTACTGAATCCTAGTAGCCAA -3′ | 5′-AGTATGAGATGGAGGCTAGT -3 |
| Rat | | |
| *TSC2* | 5- GACTCCGCCACATTAAGCGT-3′ | 5′- AGCTGGGACTTTGGTATGGG-3′ |
| *ND1* | 5′-CCGTTTTCGATATGACCAACT -3′ | 5′- TGTGTAGGGTGGAATTCCTG-3′ |
| *ND2* | 5′-CCTCACCATATTCCCAACCA -3′ | 5′- GAGGAAAGCGGTAGGGTAAG-3′ |
| *ND3* | 5′-CCCATGAGCGATTCAAACAACC -3′ | 5′- TTCATTCGTAGCTTAGGCCAAGAG-3′ |
| *ND4L* | 5′- CAGCAGTAGGTTTAGCCTTAC-3′ | 5′- TTGAGGTTTTGTACGTAGTCTG-3′ |
| *ND4* | 5′- CAACGAGGAAAACTAACCAG-3′ | 5′-TGTGATGAGTTTAGGGTTGA -3′ |
| *ND5* | 5′-CCCACCAATTATACACCGAA -3′ | 5′-TGGGATTGTCTTTTCTAGTCAG -3′ |
| *ND6* | 5′- ACTATTAAGCACCCAATACATCCAC-3′ | 5′- GTTGGCGTTGAAGCCTTCAC-3′ |
| *COX1* | 5′- GCCTTCGCATCAAAACGAGA-3′ | 5′-AGGTTCTTCGAATGTGTGGTAG -3′ |
| *COX2* | 5′- AGCTACAGTGACATCAAACC-3′ | 5′-CAATGGGTATGAAGCTGTGA -3′ |
| *COX3* | 5′- GCTCAACTTTCCTAATTGTCTG-3′ | 5′- CAAACTACATCTACGAAGTGTC-3′ |
| *atp6* | 5′- CTTGAATTTGCCGTAGCCTT-3′ | 5′- GTTATCATGTAGGTACAGGCTTAC-3′ |
| *atp8* | 5′- TTCTTCCCAAACCTTTCCTG-3′ | 5′- GAGGCAAATAGGTTTTCGTTC-3′ |
| *cytob* | 5′-ACCTCCTAATCTTAACATGAATCGG -3′ | 5′- AAAAGTAGCTGATGGAGGCTAGT-3′ |
